# Assessing the reliability of the Laban Movement Analysis system

**DOI:** 10.1371/journal.pone.0218179

**Published:** 2019-06-13

**Authors:** Ulysses Bernardet, Sarah Fdili Alaoui, Karen Studd, Karen Bradley, Philippe Pasquier, Thecla Schiphorst

**Affiliations:** 1 School of Engineering and Applied Science, Aston University, Birmingham, United Kingdom; 2 Université Paris-Sud, CNRS, Inria, Université Paris-Saclay, Orsay, France; 3 Laban/Bartenieff Institute of Movement Studies, New York, New York, United States of America; 4 University of Maryland, College Park, Maryland, United States of America; 5 School of Interactive Arts and Technology, Simon Fraser University, Vancouver, Canada; University of Glasgow, UNITED KINGDOM

## Abstract

The Laban Movement Analysis system (LMA) is a widely used system for the description of human movement. Here we present results of an empirical analysis of the reliability of the LMA system. Firstly, we developed a directed graph-based representation for the formalization of LMA. Secondly, we implemented a custom video annotation tool for stimulus presentation and annotation of the formalized LMA. Using these two elements, we conducted an experimental assessment of LMA reliability. In the experimental assessment of the reliability, experts–Certified Movement Analysts (CMA)–were tasked with identifying the differences between a “neutral” movement and the same movement executed with a specific variation in one of the dimensions of the LMA parameter space. The videos represented variations on the pantomimed movement of knocking at a door or giving directions. To be as close as possible to the annotation practice of CMAs, participants were given full control over the number of times and order in which they viewed the videos. The LMA annotation was captured by means of the video annotation tool that guided the participants through the LMA graph by asking them multiple-choice questions at each node. Participants were asked to first annotate the most salient difference (round 1), and then the second most salient one (round 2) between a neutral and gesture and the variation. To quantify the overall reliability of LMA, we computed Krippendorff’s α. The quantitative data shows that the reliability, depending on how the two rounds are integrated, ranges between a weak and an acceptable reliability of LMA. The analysis of viewing behavior showed that, despite relatively large differences at the inter-individual level, there is no simple relationship between viewing behavior and individual performance (quantified as the level of agreement of the individual with the dominant rating). This research advances the state of the art in formalizing and implementing a reliability measure for the Laban Movement Analysis system. The experimental study we conducted allows identifying some of the strengths and weaknesses of the widely used movement coding system. Additionally, we have gained useful insights into the assessment procedure itself.

## Introduction

Through movement, humans act upon and perceive the world. Given its fundamental importance, movement is studied and applied in many scientific, technological, and artistic areas. Cognitive science, for example, highlights the role of movement in cognition [1], while Human-Computer-Interaction starts to embrace the concept of embodiment [2] and the importance of movement in interaction [3,4]. Beyond its direct effect on the physical world, movement conveys intention and emotion [5]. This expressive and communicative aspect is studied in artistic domains such as dance and music, in the field of non-verbal communication, and as a means of symbolic communication e.g. through sign language. An important component of the study of movement is the development of movement classification [6], coding [7], and interpretation systems [8].

The Laban Movement Analysis (LMA) system, originally developed by Rudolf Laban in the context of performing arts [9], has been recognized and applied in numerous fields and particularly when dealing with movement creativity and computation [10,11]. LMA is an empirical observational and analytical system based on knowledge acquired through somatic and embodied practice. Though LMA has emerged from movement observation in dance, it has been applied to numerous other domains such as factory labor, robotics, and therapy [12]. Here we present our investigation into the reliability of LMA in representing and qualifying movement by assessing the consistency of LMA within and between different expert encoders. This is distinguishable from the investigation of validity that assesses the inferences that LMA allows to make, e.g. about states or traits of the observed mover. Reliability plays a pivotal role insofar as the assumption that LMA is reliable builds the foundation on which studies of validity, cognition, and the application of LMA in a technological context rest. Our study aims to establish a basic yet fundamental knowledge about LMA as a measurement instrument and should allow us to understand the value as well as the limitation of the use of such a framework in a technological and quantitative research context.

### Movement classification, coding, and interpretation systems

Given the importance of bodily movement in nearly all aspects of human existence, it is not surprising that a number of systems have been devised that allow for the translation of an observed bodily configuration or movement into a symbolic representation. What distinguishes such notation, coding, or analysis systems is their intended purpose; replay or analyze the movement or make inferences about the mover. Though many of the systems have their historical origin in an age where visual or kinematic recording was not possible, they have proven to be useful even now that these recording techniques are available.

The four major systems originating from the dance domain, and still in active use, are Labanotation/Motif [13], Laban Movement Analysis [14], Benesh Movement Notation [15], and Eshkol-Wachmann Movement Notation [16].

Originating from the closely related domains of anthropology, ethology, and psychology, are the Facial Action Coding System (FACS) [17], Kinesics [18,19], the Bernese system [20] and most recently, the Body Action and Posture Coding System (BAP) [21] and the Common Morphokinetic Alphabet (CMA) [22].

Dael et al. [21] distinguish different kinds of measurement by the degree of subjective inference ranging from subjective judgement to the use of systematic labels/observational coding to direct measure of the muscular production process. As the second criterium within observational coding systems, they identify focus on movement quality e.g. in Labanotation, Bernese systems, compared to focus on movement type e.g. in Kinesics.

Another distinguishing feature of the coding systems is their level of “granularity” along three dimensions; firstly, the smallest time unit of encoding, secondly, the number of different codes, i.e. the size of the alphabet, and thirdly, the number of tracks coded in parallel, e.g. the number of body parts. The smaller the time unit and the larger the alphabet and the number of tracks, the more fine-grain a system is. The granularity has a direct impact on the time the coding takes, i.e. the ratio of stimulus length to coding duration; fine-grain systems are very labor intense to code, while coarse-grain systems are much faster. The second aspect of the granularity relates to the subjective inference mentioned above; the finer the granularity, the less room there is for subjective judgements, and conversely, the less grainy the coding system is, the more it relies on interpretations by the coder.

At the end of the most granular system, we find the Body Action and Posture Coding System (BAP) that consists of 141 behavior variables that can be combined and that encodes time-locked temporal behavioral segments. Dael et al. [21] report in their reliability study that it took 38 hours (2,280 minutes) to encode the entire data set of 6.28 minutes. This amounts to a ratio of stimulus length to encoding duration of 1:362. For a comprehensive coding using the Facial Action Coding System, Cohn, Ambadar, & Ekman [23] report a coding ratio of 1:100, “depending on the density and complexity of facial actions”. This system consists of 44 combinable Action Units that are coded at five levels of intensities. Birdwhistell’s Kinesics system includes both fine-grained, “kineme” units of movement that are conceptually akin to phonemes, as well as movement quality descriptors. The formal system, dubbed “Kinegraph”, captures movement and configurations of whole-body and body parts and comprises hundreds of codes for head, face, trunk, shoulder/arm/wrist, neck, hand/finger, hip/leg/ankle, and foot/walking. Labanotation encodes the duration of movement for 27 different directions and levels of the movement, 18 body parts that do the movement, as well as three rhythmic patterns, 8 dynamic qualities of the movement and 6 shape qualities.

With over 65 codes, the Laban Movement Analysis system is a moderately grainy system. Our own study showed that the coding duration is highly variable; for a stimulus of about 10 seconds the coding duration is generally in the range of minutes, hence of a coding ratio in the order of 1:30. For other systems such as Kinesics, Bernese, Labanotation, or Benesh Movement Notation, published information about the ratio of stimulus length to encoding duration is not readily available.

Last but not least, some systems have been empirically assessed for their reliability (e.g. FACS [24] and BAP [25]), some to a limited extent (e.g. LMA [26]), and others to our knowledge not at all. However, high reliability is one of the most important and basic features of a coding system; if the agreement between multiple coders is low, any inference that is made based on that system is unreliable as it depends on the individual that encoded that observation.

**Fig 1:**
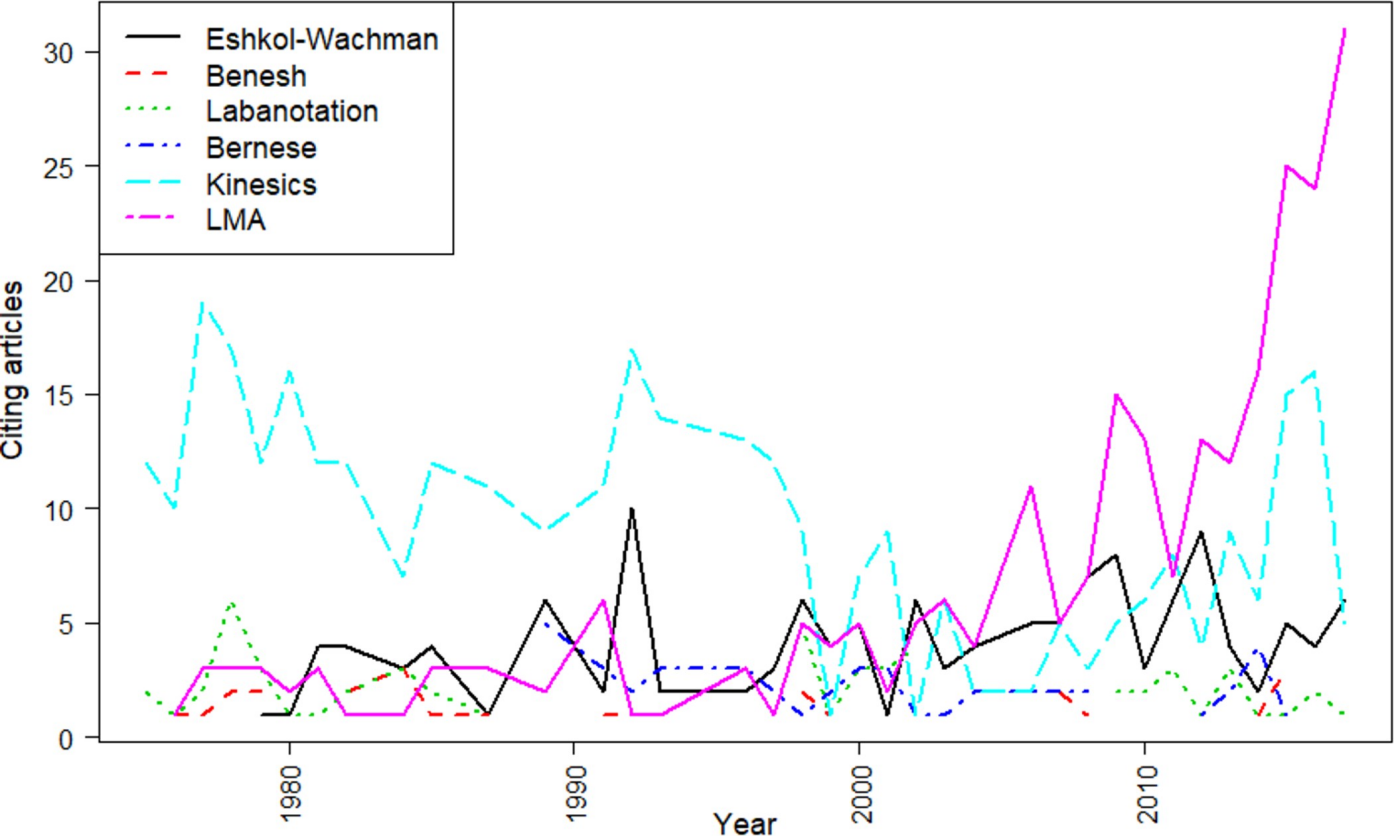
Number of articles citing the different movement coding systems. Data retrieved through Cited Reference Search using Clarivate Analytics Web of Science (WoS), data processed with the bibliometrix package for R [27].

To better understand trends in the level of adoption of the different whole-body coding systems, we analyzed how many works cite the original articles referring to the respective systems. Using Clarivate Analytics Web of Science [28], we performed a “Cited Reference Search” for the original key publications. As Fig 1 shows, the two outstanding systems are Birdwhistell’s Kinesics and Laban Movement Analysis. While the prior yielded more citations overall, the latter has clearly gained adoption in recent years.

**Fig 2:**
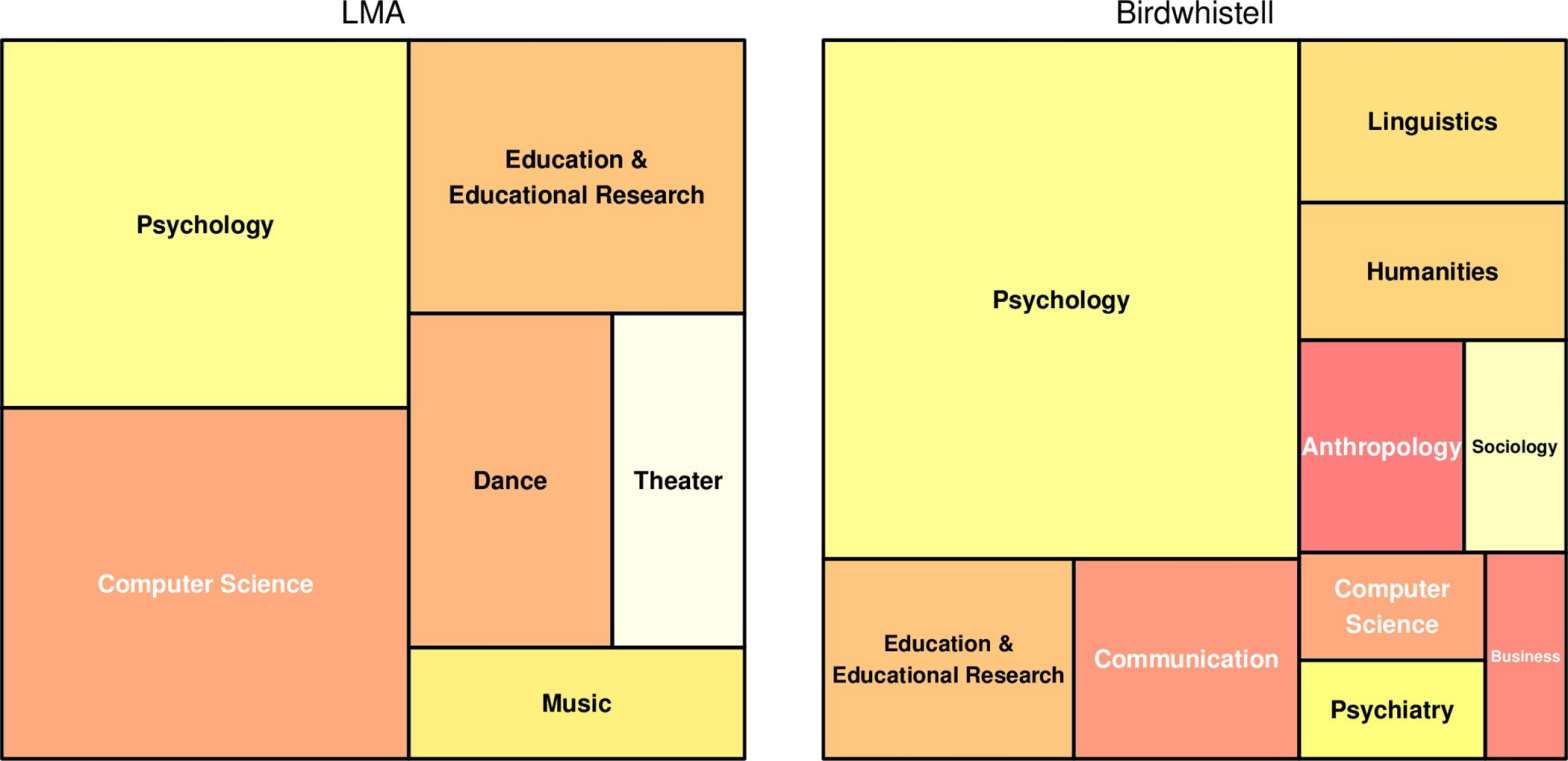
Treemap diagram of the Web-of-Science-Categories for the Laban Movement Analysis system (left) and Birdwhistell’s Kinesics (right). Entries with less than 10 entries were removed for clarity. Only the first category for each publication was considered.

Based on the same data, we further analyzed in which domains these two systems are used most. The treemap diagrams (Fig 2) show that psychology is by far the most prominent field for the application of the Kinesics system. In comparison, Laban Movement Analysis has an equally strong presence in psychology and computer science.

## The Laban Movement Analysis system

Laban Movement Analysis has a rich history. It was invented by Rudolf Laban, a movement theorist and choreographer [9] and has been applied to various disciplines including psychology [29–31], health [32], sports [33] and STEM areas such as Human-Computer-Interaction [34–36], Human-Robot-Interaction [37,38], and robotic control [39,40]. LMA bridges theory, experience and movement knowledge representation. For example, Gross, Crane, & Fredrickson [41] have used the LMA Effort and Shape framework to map the expression of emotions. Levy and Duke [42] adapted the LMA framework to the study of emotional state, personality, and movement style.

LMA provides a system to describe the function and expression of movement. In LMA, movement is observed as a pattern of change that occurs in terms of four components, defined as *Body*, *Effort*, *Space* and *Shape* (referred to collectively as *BESS*). Additionally, LMA defines the meta-category of *Phrasing*. What LMA does to understand movement is to observe, recognize and describe patterns of change.

The *Body* category describes the body parts and their actions responsible for the movement, where gestures are a sub-category of body actions. In this study, we are only investigating the reliability of LMA according to video recordings of specific gestures. In these movements, we do not vary any aspects of the *Body* category and thus will not consider Body as one of the annotation categories.

**Fig 3:**
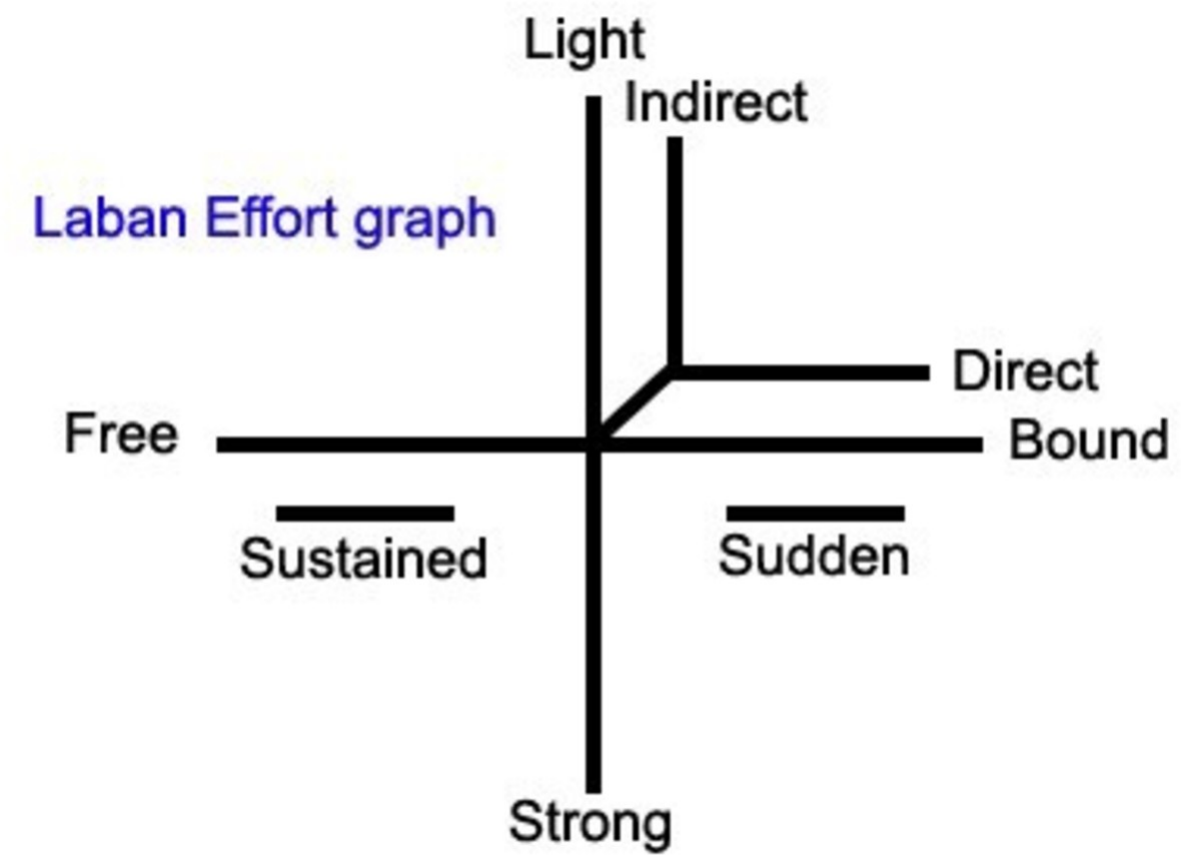
The Laban Effort continuum with the 4 Factors and their contrasting elements; Space: Direct/Indirect, Time: Sudden/Sustained, Weight: Light/Strong, Flow: Bound/Free.

LMA considers *Effort* as what can be observed and experienced in terms of the shift in attitude that reveals the mover’s attitude and intent, as well as how the mover exerts and organizes his or her energy. As such, Effort is an embodied cognitive process that initiates a process of decision-making in response to the environment [43,44]. *Effort* encompasses four factors: Weight, Time, Space, and Flow [43]. Space is related to how the mover orients his or her attention to the environment. The mover’s sense of urgency is encoded with the Time factor, while weight encodes the mover’s impact on the world. Flow captures the mover’s attitude towards bodily control. Each Effort Factor is a continuum with two opposite ends referred to as “Elements” (Space: Direct/Indirect, Time: Sudden/Sustained, Weight: Light/Strong, Flow: Bound/Free). while “Effort qualities” indicate where a movement lies on the continuum between these poles (Fig 3).

Laban formalized the *Space* component by dividing what he called the “Kinesphere”, i.e. the volume defined by the reaching possibilities of the limbs in the 3-dimensional Cartesian space with oneself at its center. We can move in a *Far Reach Space* using large movements in space, in *Near Reach Space* by moving close to ourselves, or in between (*Mid Reach Space*). Laban also defined different zones in the Kinesphere in which movement can occur: *Up*, *Down*, *Forward*, *Backward*, *Side-Open* and *Side-Across*. There are additional aspects of the *Space* category relating to Directions, Pathways, Spatial Tensions and general space, but they are not be included in the LMA notation used in our study.

*Shape* describes the change of the body’s form. Within the Shape category, our study focuses on the *Shape Qualities* that are related to the sensation, experience, and articulation of the Inner Space of the Body. *Shape Qualities* can be described with a horizontal change (*Spreading* or *Enclosing*), a vertical change (*Rising* or *Sinking*), or a sagittal change (*Advancing* or *Retreating*).

A fundamental aspect of movement patterning is the phrasing and rhythm of action. *Phrasing* emphasizes the relationship of the parts to the whole. This category looks at what aspect is emphasized in movement and how this contributes to its perceived meaning. It corresponds to where the emphasis is placed in the phrasing of the movement. *Impulsive Phrasing* encodes an emphasis at the beginning of the phrase, while *Swing Phrasing* denotes an emphasis in the middle of the phrase. An emphasis in the conclusion of the phrase corresponds to *Impactive Phrasing*.

### Observation practice in Laban Movement Analysis

The observation practice of LMA is an “embodied” practice. This means that during the movement analysis, the observer is focusing on their internal physical perception. The underlying principle is to observe the self in order to observe the other. This process leverages on the observer’s “kinesthetic empathy” and ability to relate to their own body [45]. The common coding theory posits that there exists a shared representation of perception and action [46]. This theory has received a major boost with the discovery of the “mirror neuron system” (MNS)–a set of neurons that are engaged in action observation as well as action execution [47,48]. The mirror neuron system is used to explain a wide range of phenomena including “mind-reading” [49] and empathy [50]. Studies have shown that both movement execution expertise [51,52], as well as observation expertise [53] enhance activity in the MNS. This empirical and theoretical neuronal link between movement experience and observation lends support to the LMA approach of extracting expressive qualities of movement through embodied kinesthetic observation and perception.

Fdili Alaoui et al. observed Certified Movement Analysists (CMAs) and showed that their analysis practice consists of characterizing movement as change, by comparing the observed movement to a neutral version of it [45]. This neutral version is usually imagined or enacted through the CMAs own bodies. Such embodied technique of “using the lens of the self” allows the CMAs to isolate the variation in order to better characterize it with LMA categories and labels.

### Reliability of the Laban Movement Analysis system

When using the LMA system as an observational instrument, it is essential to assess the inter-rater reliability before the system is accepted as a method for analyzing movement. To our knowledge, to date no study has assessed the inter-rater reliability for LMA as a whole.

In the study of McCoubrey [54], expert LMA analysists rated a 50-second clip of cello performance. McCoubrey focused on LMA’s Effort reliability and reported significant inter-rater reliability for the Effort factors of weight, space, and time. However, free (flow), indirect (space), and sustained (time) did not reach statistically significant inter-rater reliability. The authors also reported that most of the participants expressed frustration and confusion with the task and the inability to view the film as much as they needed, unlike what they would do ecologically.

Davis [26] investigated the reliability of Effort and Shape observations for solo dance and talk footage. However, the study had methodological limitations regarding the coding software and the selected stimuli. Teams of three CMAs, mainly dancers and dance therapists, analyzed 45 seconds of video clips of dance and people talking. Results of the study showed an inter-rater reliability for the Effort elements of strong (weight), direct (space), and sudden (time). For the dance video clips reliable agreement was obtained for the observations of sustained (time) and light (weight), as well as for the frequency of shape observations. However, for spatial direction (e.g. vertical, horizontal, sagittal) reliability was found to be poor. Davis incorporated Effort and Shape elements into a system called The Davis Nonverbal Communication Analysis System (DaNCAS) that is aimed at assessing movement behavior in the context of individual psychotherapy. After studying the coding sheet, raters were instructed to mark the presence of Effort qualities that appeared in the movement at any time during the viewing periods. The distinctions between postural and gestural movements, body parts, and degrees of intensity was *expressis verbis* not considered. Raters viewed whole-body video footage of the patient and therapist without sound. They made naturalistic, real-time observations of movement sequences and were free to review the footage as much as desired. In this study, Davis discussed the result by pointing to the need for more intensive training, with a recommended training period of between 15 and 30 hours.

Taken together, the abovementioned studies offer some limited support for the reliability of the concepts and components of LMA. However, from one study to another, a large variability in stimuli, data analysis and conclusions on inter-rater reliability seems to be present. A reliability study of the LMA system as a whole is of fundamental importance for the meaningful computation of LMA based movement features and the computational applications. However, to this day, research seems to build its LMA based models regardless of the existence of such foundation premises. These models are only based on the assumption that the LMA framework is systematic and reliable. We believe that such a gap still needs to be addressed in LMA based research.

## Methods

### Participants

A total of 18 female participants took part in the experiment. The age range was between 27 and 63 years old (mean age 43.7). All participants were Certified Laban Movement Analysts, trained and certified by the Laban/Bartenieff Institute of Movement Studies (https://labaninstitute.org). Participants graduated from the LMA certification program between 2 and 25 years ago. The study was approved by the University Research Ethics Board (REB) of the Simon Fraser University, Vancouver, Canada. Written informed consent was obtained from the participants prior to the experiment.

### Task

The participants in the experimental assessment of the reliability, CMAs were tasked with identifying the differences between a “neutral” movement and the same movement executed with a specific variation in one of the dimensions of the LMA parameter space. To be as close as possible to the annotation practice of CMAs, participants were given full control over the number of times and order in which they viewed the videos. The LMA annotation was captured by means of the video annotation tool that guided the participants through the LMA graph by asking them multiple-choice questions at each node. Participants were asked to first annotate the most salient difference (round 1) and then the second most salient one (round 2) between a neutral gesture and its variation.

### Stimulus material

**Fig 4:**
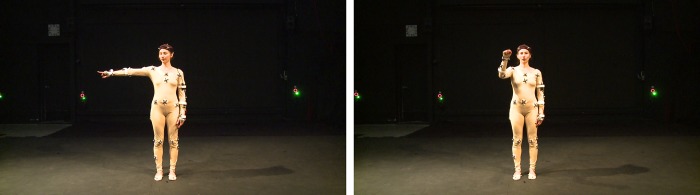
Examples of gestures “knocking” (a) and giving directions (b). (The individual in this manuscript has given written informed consent (as outlined in PLOS consent form) to publish these case details.).

To conduct this experiment, we built a database of short video clips of a dancer performing two different gesture movements in a variety of different ways. The two base gestures are *“knocking”* and *“giving directions”* (Fig 4). The gestures were chosen to be relatively simple movements that can easily be varied along the LMA dimensions of Effort, Phrasing, Shape, and Space (Table 1). Moreover, these specimens are identified as one sequence of movement that represents an individual stimulus with a single variation. The set of “*knocking”* gestures contains a total of 25 videos (mean length 3.91s): 1 neutral execution and 24 variations. The “*giving directions”* set comprises 20 videos (mean length 9.50s) of the gesture being performed with 1 neutral execution and 19 variations. The variations applied were in terms of *Phrasing* (variations in the positioning of emphasis in the phrase), *Effort* (variations in the qualities of the performed movement), *Space* (variations in the zones and reaching of the movement in the Kinesphere), and *Shape* (variations in the qualities of the change in shape). During the recording of the video stimuli, the performer was guided by a senior CMA and lead of the training program at the Laban Institute of Movement Studies. To ensure that the performer was able to achieve the various qualities of movement required to execute the LMA variations, the CMA designed a set of instructions that she gave to the dancer during the recording session. The recording process resulted in a set of videos of movement sequences that are labeled with the intended LMA characteristics.

**Table 1:** Variations of the videos in the “knocking” set.

Effort	Phrasing	Shape	Space
bound	impactive	advancing	far reach
direct	impulsive	enclosing	mid reach
free	swing	opening	near reach
light		retreating	side-across
quick		rising	side-open
strong		sinking	backwards
sustained			down
			up
7	3	6	8

We recognize that in nature it is rare to observe a movement in a “neutral” as well as a “variation” form. The rationale for our experimental design is as follows; firstly, we aimed to stay close to the practice of CMAs who characterize the movement they observe as a change (or a variation) applied to a neutral version that they enact or imagine. As CMAs observe the changes in the movement, rather than the action itself, we provide a stimulus that highlights and fixes the action through the invariant gesture. Secondly, CMAs often observe movers over a longer period of time, e.g. by being present in multiple rehearsal sessions. This effectively allows the CMA, by averaging over a longer observation period, to build a “neutral” reference model of a mover.

With the choice of the stimulus material, we seek to strike a balance between generalizability and experimental control and to build upon existing research on movement and gesture studies [6]. The trade-off is that diversity of stimuli will decrease the power of the inferential statistics because we have fewer data points for each individual stimulus. Similarly, in the rating procedure we aim at balancing efficiency and ecological validity; while we want to efficiently collect as many ratings as possible, we want to make sure that the time spent on each rating is comparable to the amount of time experts routinely allocate.

In order to ensure an exhaustive investigation of the reliability of the LMA system, we curated our database according to the categories. This will allow to assess the reliability of each LMA category in isolation. Conversely, using un-curated stimuli that come from everyday life would not allow an exhaustive assessment of LMA’s reliability. Additionally, it would be more difficult to assess the ground truth of each stimulus with regards to the existing labels. Finally, if CMAs observed un-curated stimuli, their LMA analysis would likely consist of more than 2 rounds of significant change. This would affect the experimental control over the study and jeopardize the statistical analysis through Krippendorff’s α.

It should also be noted that, in the present case, in contrast to most other publications on notation systems, we are not proposing a novel system, and demonstrating its advantages, but rather investigating the reliability of a widely used system. We therefore aim to assess the reliability of the system under optimal conditions rather than at its limits.

The videos, as well as the associated motion capture data, is publicly accessible through the MoDa Open Source Movement Database (http://moda.movingstories.ca/).

### Apparatus

**Fig 5:**
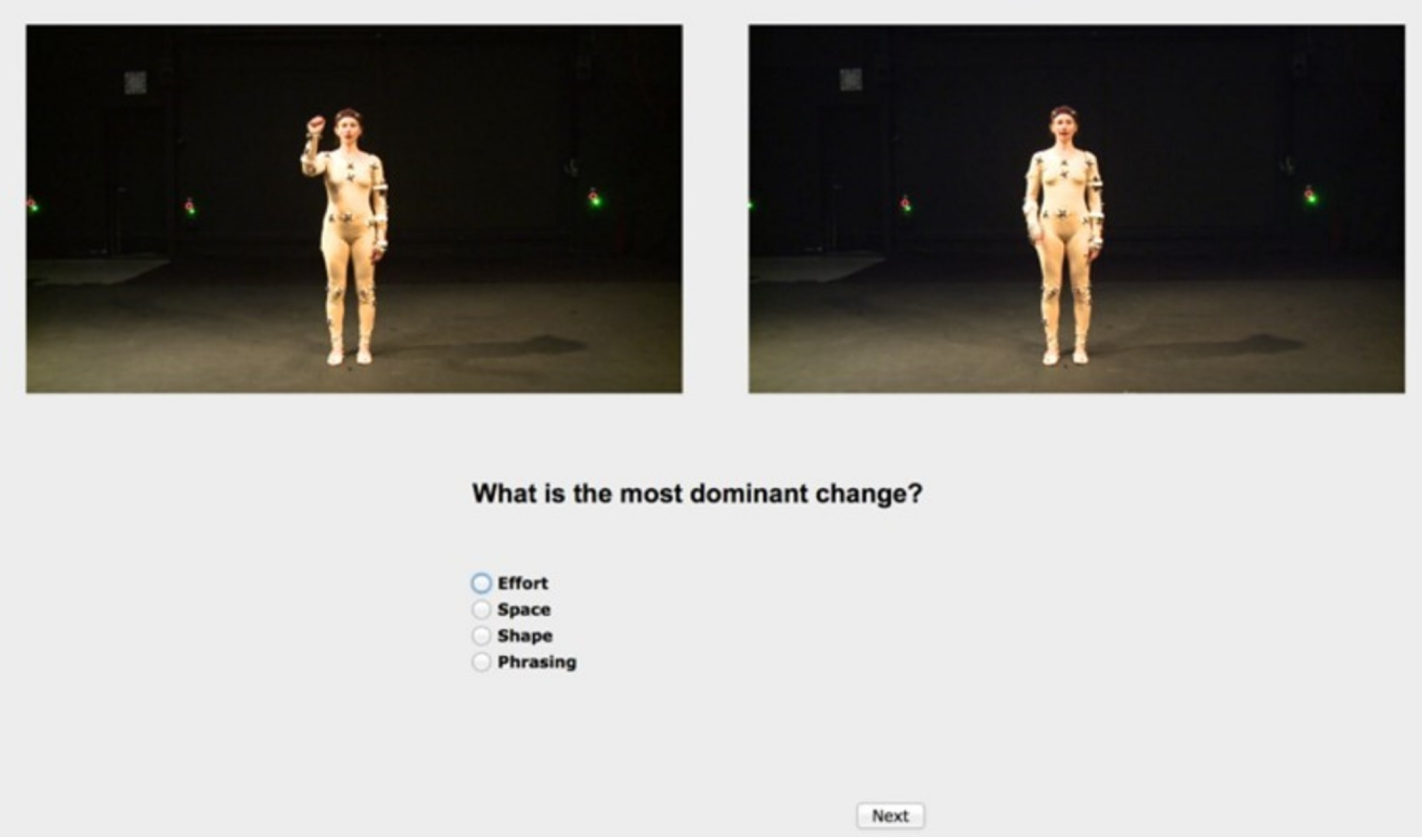
Screenshot of the video annotation tools used in the study. Participants could press play to view either video individually, or to view them both at once. (The individual in this manuscript has given written informed consent (as outlined in PLOS consent form) to publish these case details.).

For the stimulus presentation and the annotation of the formalized LMA, we developed a custom video annotation tool (Fig 5). The tool was developed iteratively in close collaboration with four CMAs. A number of tools are available for annotating videos, both commercial (Mangold interact, https://www.mangold-international.com/en/products/software/behavior-research-with-mangold-interact), Noldus Observer XT, https://www.noldus.com/human-behavior-research/products/the-observer-xt), and open source (ELAN, https://www.mpi.nl/corpus/html/elan/index.html, Kinovea, https://www.kinovea.org/, Anvil [55]). However, most of these tools are geared towards annotation along a time line, and none of them support a decision-making process based on a directed graph. The novelty of our LMA video annotation tool is that the user is guided through a set of questions and answers that are based on the underlying directed graph representation of LMA. The graph itself is stored in a database where one table maps the questions to possible answers, and a second table "A2Q" maps answers to follow-up questions. By changing the underlying database, the annotation tool can readily be adapted to support any directed graph type of decision process. The primary purpose of the LMA video annotation tool is to support the decision-making process during the LMA annotation process. To make the tool accessible to the wider LMA community, we have released the software under the GNU GENERAL PUBLIC LICENSE Version 3 on GitHub (https://github.com/bernuly/LMAVideoAnnotionTool).

### Coding procedure

From a total of 25 videos of *knocking* and 20 videos from the *giving directions*, we randomly selected 12 *knocking* and 10 *giving directions* videos. Each participant was asked to annotate a pair of videos of either knocking or giving direction gestures: the neutral gesture and a variation of it. Note that the neutral video itself was included in variations. Hence some of the comparisons were neutral with neutral. The pairs were presented in a randomized order. Each participant annotated a total of 22 pairs of videos. For each annotation, participants were presented with two videos: The video of the gesture being performed as neutral was presented on the left. The video of the gesture with the variation was presented on the right. Therefore, the participants were asked to annotate the change observed from the neutral to the varied movement.

**Fig 6:**
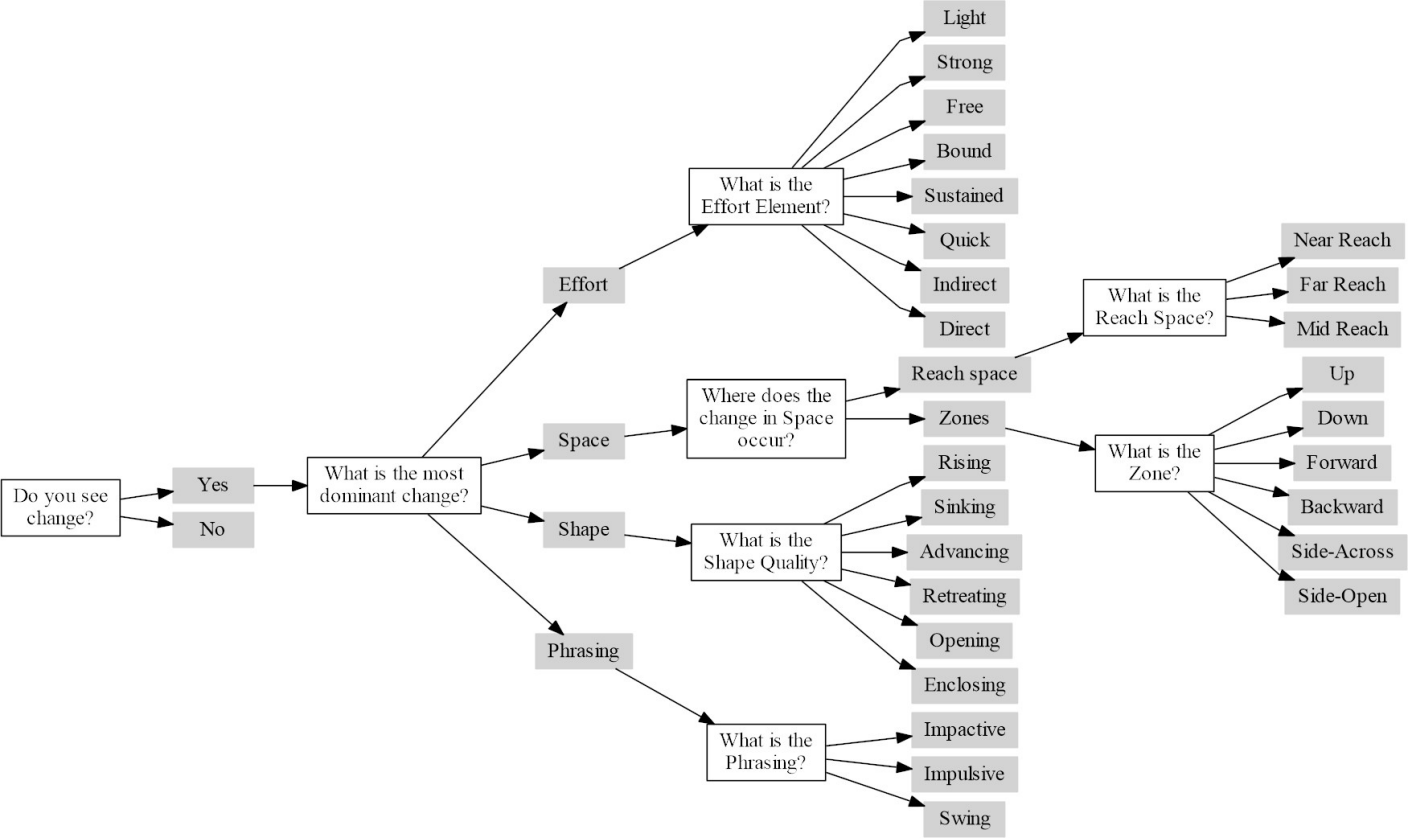
Decision tree on which the LMA annotation is based.

After having viewed both videos at least once, the participants were presented with a questionnaire of multiple choices answers based on the decision tree presented in Fig 6. This decision tree was designed iteratively with the four CMAs that took part in the experimental design. It corresponds to how the categories of Effort, Space, Shape and Phrasing are organized in LMA. The first branch of the three characterizes the change into one of the four LMA categories of Effort, Space, Shape and Phrasing. The second branch characterizes respectively the Effort elements, the reach and zones in Space, the Shape qualities and the Phrasing patterns. The participants were asked a series of questions leading to an LMA annotation. The tree had a maximum depth of four levels. Note that for the analysis, we only take the endpoint of each branch into account, which results in a total of 27 possible annotations.

After having completed one iteration of the decision tree, participants were given the option of annotating a second change that they had observed. The second annotation followed the same procedure as the first one. The system did not enforce that the second annotation had to be different from the first one. When the video clips were created, the intention was that each clip only contained a single LMA variation. We (including the four CMAs) designed the tool to provide two choices of the most and the second most significant changes observed in movement. Indeed, it is an integral part of LMA to recognize that variations rarely occur in isolation. For example, a change in Phrasing might be achieved through an emphasis in Effort at the beginning or the end of the phrase. Another example is the affinities of Effort with Space directions or Shape qualities. In the latter, the two variations occur simultaneously. Lastly, Effort often occurs in pairs and not in isolation. The consequence of this is that CMAs might see the same changes but give them different prioritization. To accommodate for this in the context of movement change annotation, we asked participants to annotate the movement twice: Their first choice is the most significant change, then they choose the second most significant change.

## Results

### Calculating reliability

To assess the overall reliability of LMA we computed Krippendorff’s α [56]. Krippendorff’s α is a statistical measure that describes the level of agreement achieved when coding units of analysis, a generalized measure of inter-coder agreement or inter-rater reliability. It is used in psychological experiments where there is a need to compare tests of the same phenomenon. In observation studies, it is used when unstructured coding is recorded for subsequent analysis. This measure is applicable to any number of coders, to incomplete data, to any number of coding values, and to various types of metrics (binary, nominal, ordinal …etc.) [57]. The advantage of such a single coefficient is that it represents a degree of reliability that can be compared across any number of coders, values, metrics, and unequal sample sizes.

Calculating Krippendorff’s α consists of three main steps: calculation of the observed coincidences (which yields a “coincidence matrix”), application of the distance function to the matrix, and calculation of the overall α value [56]. The calculation of the coincidence matrix is a core feature in calculating Krippendorff’s α. The matrix cross-tabulates the n pairable values (pair-wise combinations across all participants) into a v-by-v square matrix, where v is the number of levels of a variable [56]. The coincidence matrix is symmetrical around the diagonal, and if all values are matching perfectly, all coincidences fall on the diagonal.

**Fig 7:**
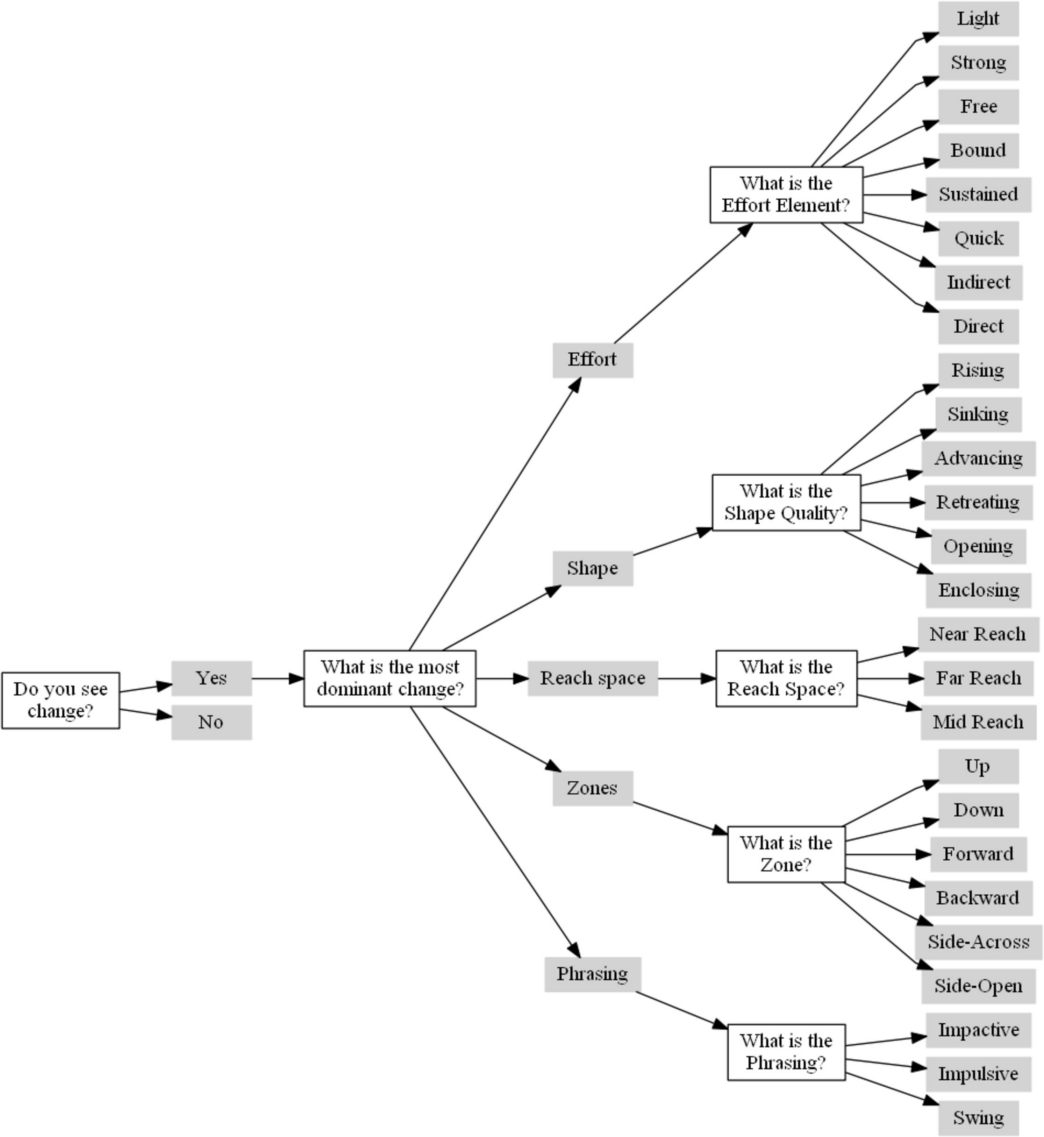
LMA graph with equal lengths to the final answer.

The next step is applying the distance function to the coincidence matrix. Choosing the function to compute the pairwise distance between any two answers is one of the key decisions in calculating the α value. Note that in the original graph, the length of the paths to the last answer is not the same for all branches; e.g. to arrive at the answer “Near Reach” it takes 4 questions and answers, while getting to the answer “Rising” takes only 3 questions and answers. Hence, to unify the length of the paths, we remove the answer “Space” and the subsequent question “Where does the change in Space occur”. The trimmed graph is showed in Fig 7. As shown in Eq 1, we use a difference measure that is based on the number of overlapping answers in the answer sequences that represent the specific path on which participants traversed the directed LMA graph (Fig 6).

$${Difference}_{\left(sequence,sequence\prime \right)}=1-\frac{N_{pairs\;equal}}{N_{pairs\;total}}$$ (1)

Hence, if we compare two sequences that each comprise four answers with two matching pairs (e.g. “Yes-Effort-Light” and “Yes-Effort-Strong”) this will result in $\mathrm{difference}=0.33=1-\frac{2}{3}$. Note that due to the constraint nature of the directed graph of the structure of questions and answers, we do not need to take into account where in the sequence the overlap occurred.

#### Different approaches to combining round 1 and round 2 sequences

In the measurement procedure we used in our study, each participant rated the observed behavior twice, ordered by the assessed relevance of the change. Hence, our results comprise two answer sequences per participant per pair of videos. For each round, each participant had a choice between 27 different paths. We will refer to these two sequences as “R1” and “R2”.

We computed Krippendorff’s α for four different combinations of data from R1 and R2. Firstly, we computed α only taking into account data from the first round of each participant (“R1”). Subsequently, we computed α for two methods of combining R1 and R2 data (“R1xR2”); order dependent and order independent. In the order dependent case, given that a single round offers 27 possible paths, each participant could choose between 272 different paths. Hence the coincidence matrix underlying Krippendorff’s α, in this case, had a size of 272* 272 cells. We immediately see the effect of this massive amount of possible answer combinations in the drop of α to almost half of R1 alone (Table 2). In the order independent case the R1 and R2 answers per participant were aligned such as to maximize overlap when computing the coincidence matrix. This means that if participant 1 gave sequence A in round 1 and sequence B in round 2, this was assumed to be equal to participant 2 giving sequence B in round 1 and sequence A in round 2. This analysis favored cases where the difference between participants lay merely in the prioritizing, not in the LMA assessment per se.

**Table 2:** Variations of the videos in the “direction” set.

Effort	Phrasing	Shape	Space
bound	impactive	advancing	down
direct	impulsive	enclosing	mid reach
free	swing	opening	near reach
light		retreating	
quick		rising	
strong		sinking	
sustained			
7	3	6	3

All these methods of combining R1 and R2 do not seem to reflect well the initial rationale of using two rounds of encoding, i.e. that differences between raters might be due to different prioritizations. For this reason, we chose a fourth method that maximized agreement between participants. The central idea of this combination method is to take from each participant one of the answer sequences (R1 or R2) in such a way as to maximize overall agreement between encoders, with maximization in this case meaning minimal sum distance between each answer. To ensure finding the global minimum “R optimal”, we computed the sum difference for each possible combination of all R1 and R2s for each participant. Though this approach is somewhat unconventional, it does reflect the discursive approach often taken by a group of CMAs when assessing a movement.

#### Comparing combination methods

**Fig 8:**
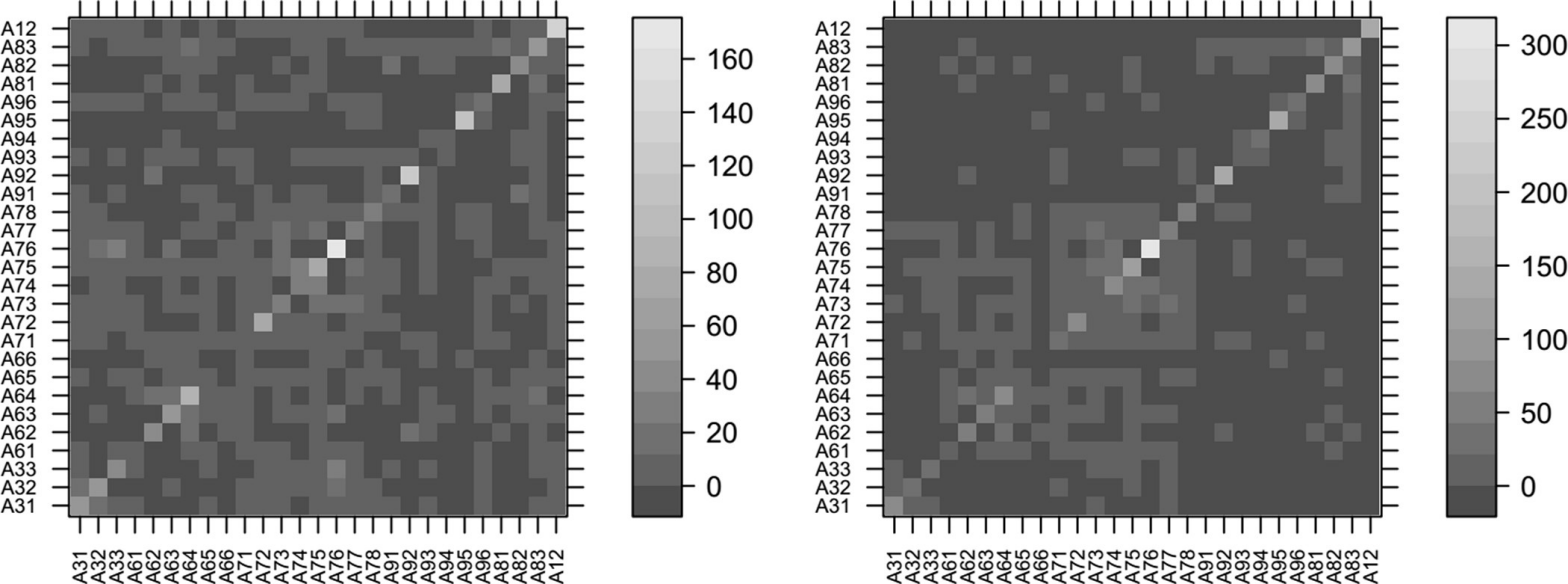
Coincidence matrices for round 1 only (a) compared to “best of” round 1 and round 2 (b). Hits on the diagonal axis indicate agreement between two raters. Note that the eccentricity from the diagonal axis does not indicate the level of disagreement since the order of the answers on the ordinate and abscissa is not necessarily corresponding to their similarity.

Even before computing Krippendorff’s’ α itself, we can see a difference between the “R1 only” and “R optimal” approach at the level of the coincidence matrices (Fig 8): The overall spread for “R1 only” is markedly larger than the one for “R optimal”.

Table 3 summarizes the α values computed using the different combination methods listed above. The table shows that neither method of combining round 1 and round two–order dependent or order independent–yielded an α higher than round 1 alone. When including both rounds, we do observe a higher α when not considering the order. Comparing round 1 alone with the “R optimal” combination strategy we see the expected marked increase of α (Table 3).

**Table 3:** Krippendorff’s α computed for different methods of combining round 1 and 2.

Subset	α
R1 only	0.473
R1xR2 order dependent	0.219
R1xR2 order independent	0.305
R optimal	0.676

The stimulus material consists of video clips in which a dancer performs two different gestures–“knocking” and “giving directions”–in a variety of different ways. The variations on the movement execution are along the LMA dimensions of Space, Effort, Phrasing, and Shape (Table 1). In a next step of analysis, we wanted to better understand the specific strength and weaknesses in what LMA can encode. In order to do so, we calculated Krippendorff’s’ α for the two gesture types on the one hand, and the different variations on the other hand. As we can see from Table 4, there is no marked difference in the reliability with which the two gestures can be encoded. A different image presents itself with respect to the variations in each category (Table 5); while Space and Phrasing are rated the most reliably, the Effort category is the most difficult one to agree on.

**Table 4:** Krippendorff’s’ α values for the gestures based on “R optimal” combination method.

	alpha
direction	0.65
knocking	0.69

**Table 5:** Krippendorff’s’ α values for the variations based on “R optimal” combination method.

	alpha
space	0.66
effort	0.46
phrasing	0.66
shape	0.50

### Viewing behavior

We can ask if any of the gestures or any of the categories or any of the questions required more viewing before being answered.

**Fig 9:**
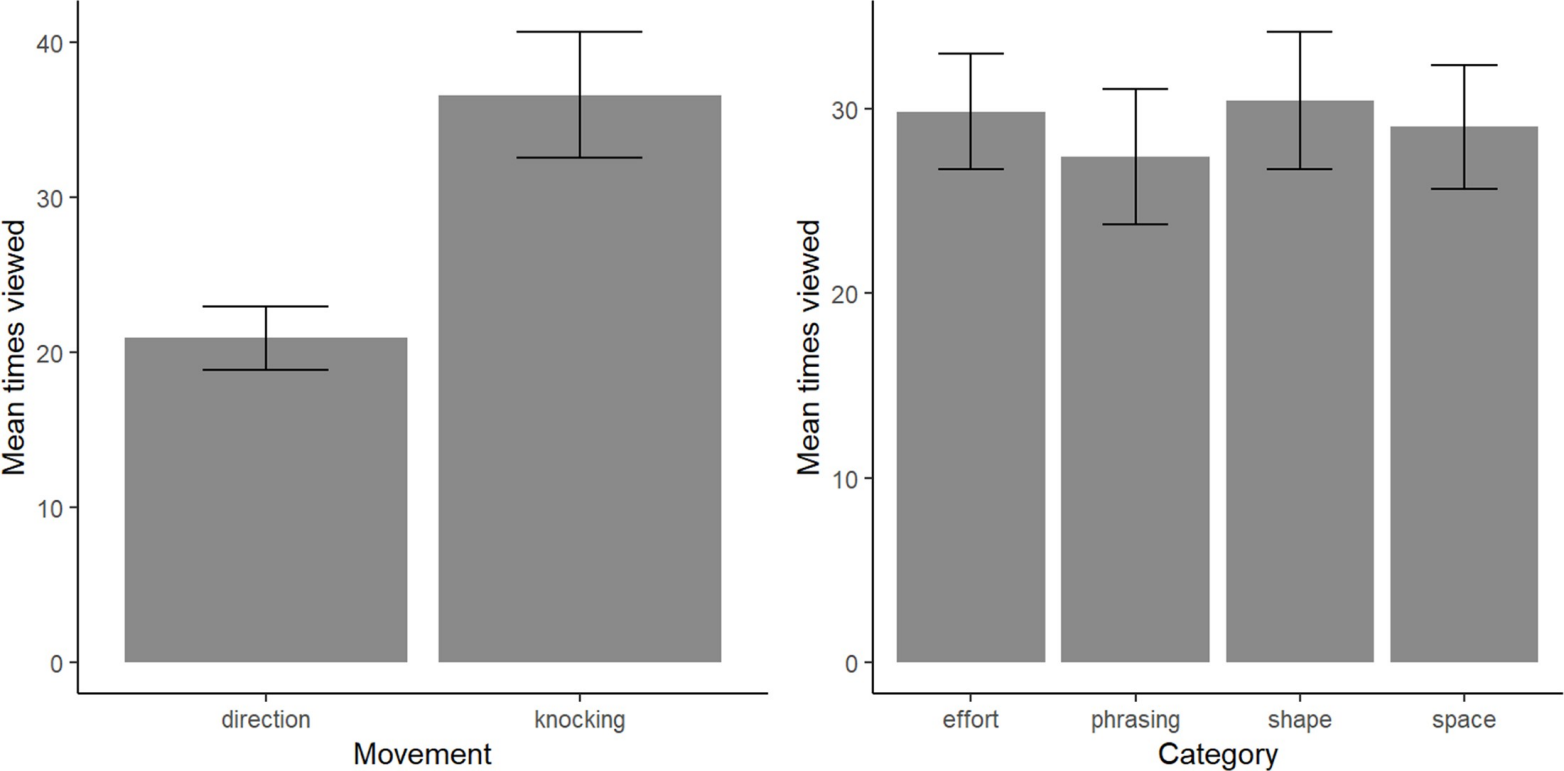
(a) frequency of viewing for the two gestures, (b) frequency of viewing for the individual categories.

**Fig 10:**
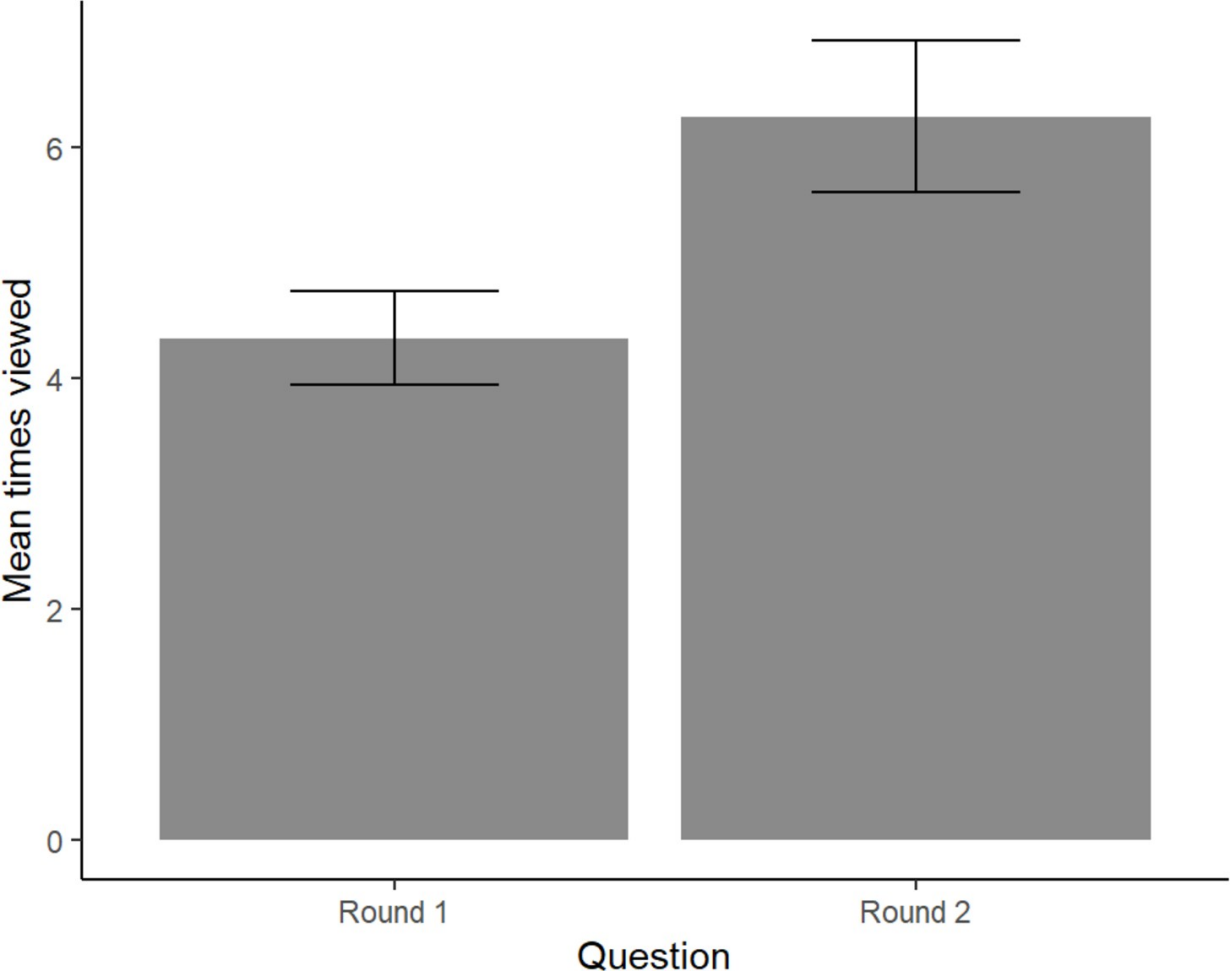
Mean number of times videos were viewed per individual question in each round.

Firstly, we looked at the difference between the two gestures. A paired-samples t-test comparing the effect of Movement type yielded a significant difference in the number of times the videos were viewed for “direction” (M = 20.93, SD = 8.682) and “knocking” (M = 36.57, SD = 17.23); *t*(17) = -5.48, *p* < .001 Fig 9A. Note that the viewing numbers show in Fig 9A are the sum over all questions, i.e. the total number of times the participants viewed the videos to answer up to 8 questions. An analysis at the level of single questions shows that the mean number of times a video was viewed per individual question is in the order of 6 times (Fig 10). To compare the effect of LMA Category on viewing frequency, we conducted a one-way between subjects ANOVA. This analysis did not find a significant effect for the four conditions (*F*(1.77, 30.03) = 0.34, *p* = .690) (Fig 9B). However, a paired-samples t-test revealed a significant difference in the mean number of times a video was viewed to answer an individual question for “Round 1” (M = 4.348, SD = 1.715) and “Round 2” (M = 6.271, SD = 2.77); *t*(17) = -5.85, *p*< .001. (Fig 10).

**Fig 11:**
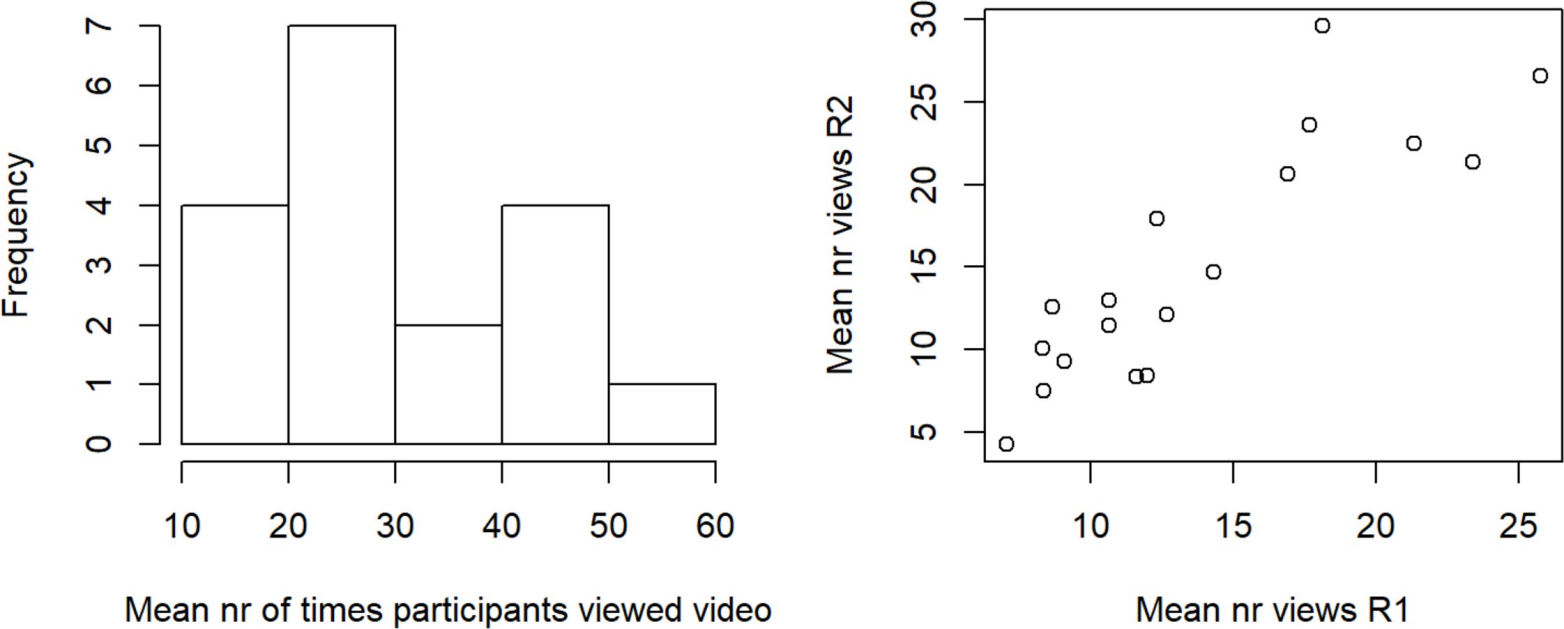
(a) Viewing frequency at the level of individual participants. (b) Relationship between viewing in the first and in the second round (Pearson’s r = 0.87, p<0.001).

While conducting the experiment, we observed that participants differed markedly in the amount of time they took to answer the questions. This observation is corroborated by the analysis of the viewing behavior at the level of individual participants. The histogram of viewing times (Fig 11A) shows that participants viewed videos anywhere from 10 to 60 times. Interestingly, there is a strong correlation between the viewing behavior in the first and second round (Fig 11B), which indicates that there are consistent individual differences.

### Individual rating differences

Krippendorff’s’ α calculated above is a single, global measure of reliability. To perform an analysis at the level of a single participant, we need a measure that quantifies the rating performance at the individual level. Subsequently, we will refer to this measure as individual “Similarity”. To compute each participant's similarity for each video, we first assessed which sequence of answers was given most often for a specific video. Based on this most frequent answer, we then computed for each participant how similar their answer was based on the overlap coefficient [58] shown in Eq 2.

$${Similarity}_{\left(most\;common\;sequence,sequence\;participant\right)}=\frac{N_{pairs\;equal}}{N_{pairs\;total}}$$ (2)

**Fig 12:**
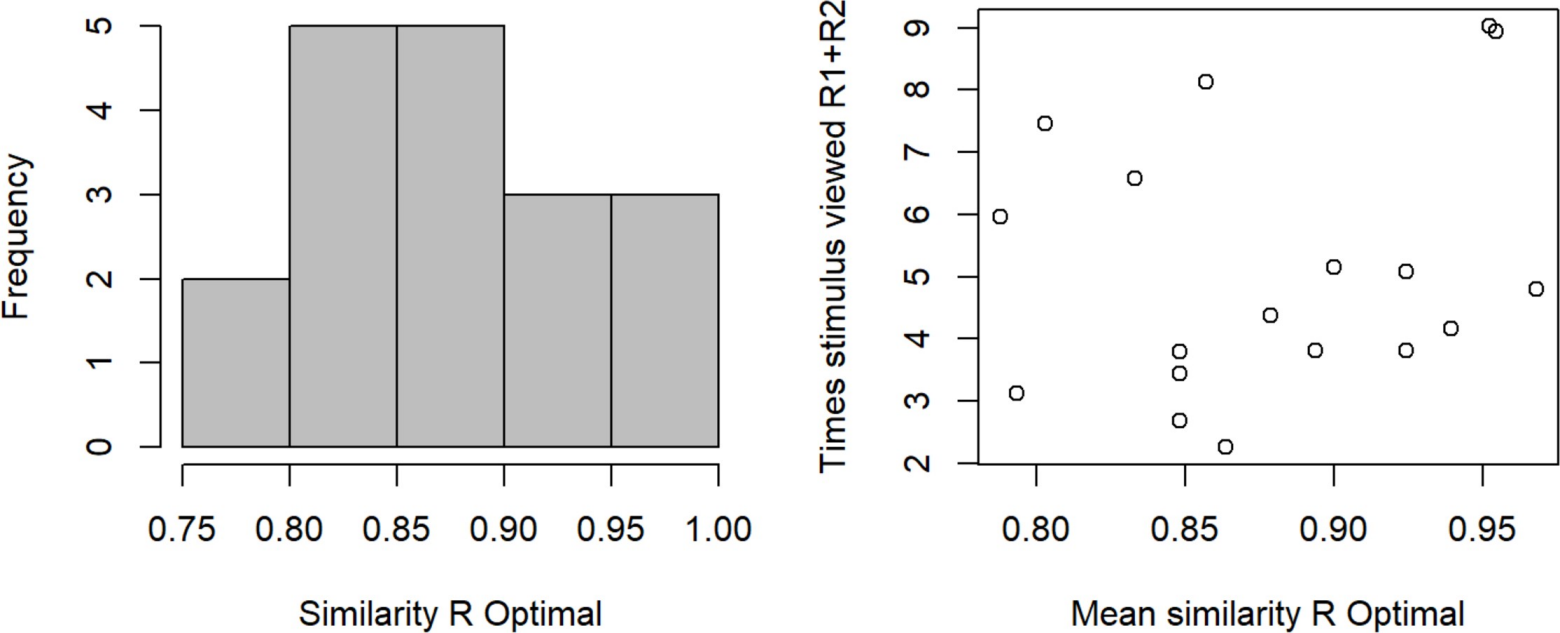
Similarity of an individual’s answer with the most common answer sequence vs stimulus video views. (a) Frequency distribution of the individual similarity (b) Mean per participant across all videos (Pearson’s r = 0.07).

The histogram of the similarity index shows that the distribution was relatively broad (Fig 12A). Additionally, the similarity measure allows us to relate individual encoding performance to other behavioral measures. Interestingly, we did not find a significant correlation between similarity score and number of times the stimulus video was viewed at the level of the individual participants (Fig 12B).

## Discussion

We present the results of an empirical analysis of the reliability of the LMA system. To do so, we developed a directed graph-based representation for the formalization of LMA, implemented a custom video annotation tool for LMA annotation of stimulus, and ran the experimental assessment of LMA reliability. In our experiment, we asked CMAs to identify the change between a “neutral” gesture of knocking or showing direction and the same gesture executed with a specific variation from the LMA parameter space. CMAs could choose the number of times and order in which they viewed the videos. They were asked to first annotate the most significant change (round 1), and then the second most significant one (round 2) between the neutral gesture and its variation. Their annotation was captured by means of the video annotation tool that guided them through the LMA graph through multiple-choice questions at each node.

To quantify the overall reliability of LMA, we computed Krippendorff’s α. The quantitative data shows that the reliability, depending on how the two rounds are integrated, ranges between a weak and an acceptable reliability of LMA. Our results also show that the categories of Space and Phrasing achieve a higher reliability than the categories of Effort and Shape. The analysis of viewing behavior showed that, despite relatively large differences at the inter-individual level, there is no simple relationship between viewing behavior and individual performance (quantified as the level of agreement of the individual with the dominant rating).

At the individual level, we found that CMAs varied both in their viewing behavior and in their performance in terms of individual agreement with the majority rating. We found the viewing style to be consistent at the level of the individual, but unrelated to performance.

While overall, we found acceptable levels of inter-rater agreement; there were marked differences between the LMA categories. The required level of subjective inference might explain the difference between Phrasing and Space on the one hand and Effort and Shape on the other hand. Phrasing and Space are considered more quantitative categories because they relate to notions of time and space that are delineated regardless of personal interpretations. The kinesphere in the Laban System defines 27 directions and the observation of the direction of a movement consists of approximating it to one of the theoretical discrete directions in the kinesphere. This usually does not relate to one’s personal signature or preference. Phrasing is also about observing where the emphasis was put in the overall timing of a phrase. Therefore, it consists of comparing the level of energy that the movement has exerted in different time frames (beginning, middle, or end of the phrase). Observing phrasing is thus less correlated with the observer’s own Phrasing patterns. Conversely, the lower reliability in Effort and Shape can be explained by the more qualitative nature of these categories of observation. In these two categories, there are no direct systematic measurements of its characteristics. They are qualitative and relate more so to the observer’s preferences, personal style and movement signature. For example, a coder whose personal signature includes Light Weight Effort could tend to observe more Lightness in people’s movement patterns. This explains the difference in the results of our reliability measure in the categories of Effort and Shape.

## Limitations

LMA is commonly used to analyze politicians, performers, and a range of other everyday life situation. Hence, in order to increase the ecological validity of the stimulus material, we decided to use a trained dancer, rather than a CMA to perform the actions in the stimulus videos. This might have affected the reliability results because we cannot ensure that the variations performed occur alone, isolated from other variations. To accommodate for this, we let the participants annotate two rounds during the experiment so that they can account for more than one change that might have occurred. However, if more than two changes occurred then it is more difficult to isolate reliably without personal biases and preferences and interpretation, which can affect the agreement among CMAs. The introduction of the second round of annotation meant that prioritization itself became a deciding factor for the reliability and not the categorizations themselves. Taking this into account, a future study might decide to let raters classify the four categories of Space, Effort, Shape, and Phrasing, rather than aim to produce initial stimulus material where the categories are isolated.

The CMAs who participated in the study reported that the annotation tool was useful in that it provided them with a way to observe and annotate videos on a single platform, i.e. without the need to switch between observing the video on a screen and writing their observations on a paper. As a point of criticism, the CMAs noted that the tool does not consider the collaborative aspect of the annotation process; to achieve a more reliable observation, analysts usually seek for consensus through group observation. This consensus process as described by Fdili Alaoui et al. [45] and is central to balancing individual personal observation with group consensus. That we did not consider consensus in this experiment limits how much our results generalize to a real-life situation where usually at least two CMAs observe together and challenge their observations to find a ground truth. Note that this is also a practice that has been generally ignored in previous experiments and that poses clear methodological challenges. We have tried to mitigate this limitation and accommodate for this consensus-based approach by combining the two annotation rounds into an “R Optimal”, maximal overlap annotation. In a follow-up study, Fdili Alaoui et al. [45] have adapted the tool for a group annotation of the movement sequences and were able to gain insight into the process of CMAs analyzing movement collaboratively through negotiation and consensus-seeking.

## Conclusion

Though few of the previous studies analyze their results using a standard reliability *measure* such as Krippendorff’s α, it seems that we found LMA to be somewhat less reliable. However, a direct comparison is made difficult because of the substantial differences with previous studies in terms of the rater sample, movement specimen, procedure, coding method, and LMA categories included. First, all approaches differ in the type of *rater*; some studies use expert movement observers and expert Laban analysts i.e. CMAs, while others use non-expert raters. Another important difference is the choice of the *movement specimen*. For example, McCoubrey [54] investigated LMA’s Effort reliability with CMAs that rated a stimulus of cello performance, while Davis [26] used dance and talk footage. Most of the inter-rater reliability studies to date have not scanned all *categories* presented in LMA. Most of them focus exclusively on the Effort and Shape category. Conversely, to our knowledge, no study has assessed the reliability of the Phrasing or the Space characteristics of movement. We used a graph-based approach that allowed us to capture most of the LMA categories in Space, Effort, Shape and Phrasing and to investigate them in isolation in order to study their reliability individually. This direct mapping offered the advantage of targeting the categories of the LMA framework directly and the fact that participants did not have to learn a new coding system as was the case e.g. when using the “Davis Nonverbal Communication Analysis System” used by Davis [26].

In its traditional practical application, LMA is a model in which practitioners learn to understand themselves as the premise for understanding others. It is a practice-based method that allows articulating movement both through acknowledging individual difference (first person perspective) as well as learning what is “objective” (third person perspective). Interestingly, literature using LMA in computational systems assumes its objectivity as a third person coding method only. On the other hand, literature using LMA as a method to enhance bodily awareness assumes its somatic and experiential values only. Taking into account the focus of LMA practice and teaching on both first- and third-person perspective mixing objective coding with subjective experience, we find an impressive level of reliability between coders. As it is learned, LMA does not guarantee a general reliability and universality. This means that to achieve a rigorous reliability, a shift would be necessary in the core philosophy and in the applications of LMA. Such a shift might be desirable when LMA is used in a computation context. In such approaches, CMAs are usually the authority that provides the ground truth against which automation is tested and to be valid, this ground truth needs to be established in a rater-independent fashion.

To our knowledge, our study is the first comprehensive, expert rater-based investigation of the reliability of the Laban Movement Analysis system. We can see that by feeding our insights back into the development of the assessment and teaching methods, the reliability and with it the utility of LMA can only increase. Additionally, we gained useful insights into the assessment procedure and the formalization and implementation of a reliability measure for the Laban Movement Analysis system. Our methodological insights can be applied to other coding systems and the wider literature on non-verbal behavior.

Future directions include studies that use a more open, less LMA-tailored dataset, the use of a deeper LMA graph, as well as the addition of collaborative features to the video annotation to better support annotation in groups.
